# Detecting Traversable Area and Water Hazards for the Visually Impaired with a pRGB-D Sensor

**DOI:** 10.3390/s17081890

**Published:** 2017-08-17

**Authors:** Kailun Yang, Kaiwei Wang, Ruiqi Cheng, Weijian Hu, Xiao Huang, Jian Bai

**Affiliations:** College of Optical Science and Engineering, Zhejiang University, Hangzhou 310027, China; elnino@zju.edu.cn (K.Y.); rickycheng@zju.edu.cn (R.C.); huweijian@zju.edu.cn (W.H.); huang_opt@zju.edu.cn (X.H.); bai@zju.edu.cn (J.B.)

**Keywords:** traversable area detection, water hazard detection, RGB-D sensor, polarized stereo, ZED, visually impaired people

## Abstract

The use of RGB-Depth (RGB-D) sensors for assisting visually impaired people (VIP) has been widely reported as they offer portability, function-diversity and cost-effectiveness. However, polarization cues to assist traversability awareness without precautions against stepping into water areas are weak. In this paper, a polarized RGB-Depth (pRGB-D) framework is proposed to detect traversable area and water hazards simultaneously with polarization-color-depth-attitude information to enhance safety during navigation. The approach has been tested on a pRGB-D dataset, which is built for tuning parameters and evaluating the performance. Moreover, the approach has been integrated into a wearable prototype which generates a stereo sound feedback to guide visually impaired people (VIP) follow the prioritized direction to avoid obstacles and water hazards. Furthermore, a preliminary study with ten blindfolded participants suggests its effectivity and reliability.

## 1. Introduction

According to the World Health Organization, 285 million people around the world are estimated to be visually impaired and 39 million of them are blind [1]. It is rather challenging for visually impaired people (VIP) to naturally navigate through obstacles and avoid water hazards in unknown environments. The invention of RGB-D sensors had a prodigious influence to the research field of visually impaired people assistance with the emergence of light-coding sensor-based approaches [2,3,4,5,6,7,8,9,10,11,12,13,14,15,16,17] and stereo camera [18,19,20,21,22,23,24,25,26,27,28,29]-based approaches as well as the proof-of-concepts they provided.

The appearance of RGB-D sensors across a wide range of visual assistance contexts is not only due to their low-power-consumption and cost-efficiency, but also its robustness and good performance as they are able to simultaneously perceive color and depth information at video framerates. Apart from color and depth, polarization and its imaging extend information dimension to be used for material discrimination and target detection because polarization parameters reflect physical properties of materials [30]. Meanwhile, given that light with different polarization state behaves differently at the interface of object surface, polarization has been applied to many surface measurement techniques. However, typical commercial RGB-D sensors including light-coding sensors and stereo cameras simply rely on intensity information whereas their polarization cues are generally unavailable or weak.

Light-coding sensors project near-IR speckles and capture the patterns with a calibrated camera, the structured light pattern being coded with spatial or temporary projecting methods [31]. These sensors correlate observed pixels with projected pixels so as to derive depth information through triangulating algorithms. However, many consumer-grade sensors are coded in intensity rather than polarization regardless of whether they use spatial or temporary coding methods. Thereby, as projected speckles are easily lost in sunlight, approaches for VIP with light-coding sensors focus mainly on obstacle avoidance [2,3,9,11,13], traversability awareness [5,6,7,8,12,14] and stairs detection [4,10,15,16,17].

A stereo camera estimates depth maps through stereo matching of images from two lenses. There is a large body of work on stereo matching, some of which are local stereo matching algorithms [32,33,34] while others approximate global optimization [35,36,37]. Local stereo algorithms are generally faster as they calculate the depth of pixels based simply on the correlation of local image patches. Most commercial stereo cameras include local algorithms to achieve speedy refresh rates but return sparse depth information in texture-less scenes, such as a blank wall. Different from conventional stereo cameras, the RGB-D sensor of the RealSense R200 and RS400 [38] devices employed active stereo [39] to combine a pair of cameras with fixed structured light patterns to realize extraordinary environmental adaptability. ZED [40] implemented GPU-accelerated global stereo algorithms to achieve dense and large-scale depth perception, but these commercial stereo cameras comprised IR or RGB lenses and none of them use polarization modulation. As a result, most approaches for VIP with stereo cameras assist navigation in large outdoor spaces [18,19,20,21,22,26,27,28,29] and some schemes have also addressed localization [23,24,25], but none of them detect water hazards to prevent misleading VIP to step into water areas.

In this paper, we proposed to use a polarized a RGB-D sensor for detecting traversable areas and water hazards by adequately considering polarization effects. We equipped a stereo camera with an attitude angle sensor, a pair of horizontal and vertical polarizers to form a pRGB-D sensor. The point cloud in 3D space relative to the sensor is computed with the depth image and intrinsic parameters. The points are adjusted to the world coordinate system with the attitude angles of the sensor. The traversable area is determined by building a stochastic polar occupancy grid, estimating the ground pose by fitting a B-spline surface and using dynamic programming to segment the ground area and obstacles. The water areas are identified by using polarization effects as the primary cue and the polarization difference image is computed with disparity information from the pRGB-D sensor.

This work extends what was presented in [11,12,29], where we addressed the traversable area detection and scene segmentation with a wearable RGB-D sensor. In this paper, we have attached the filters to add polarization information, and generated a stereo sound feedback system to take into account both traversable regions and water areas to guide VIP to follow the prioritized direction in order to avoid obstacles and water hazards, as well as set up a pRGB-D navigation framework which has been proved to be useful and reliable by a field test with ten blindfolded participants.

This paper is organized as follows: in Section 2, related works that have addressed traversability awareness or water hazard detection are reviewed; in Section 3, the presented approach is elaborated in detail; in Section 4, the effectiveness and robustness in terms of the detection and the stereo sound interface are demonstrated with experiments and a user study; and in Section 5, relevant conclusions are drawn and future works are expected.

## 2. Related Work

Several related works have been dedicated to traversable area detection, which can be divided into two categories: traversable area detection based on scene segmentation and traversable area detection based on surface normal vector estimation. Dakopoulos presented a comparative survey of wearable obstacle avoidance systems to inform the research community and users about the capabilities of the progress in assistive technology for VIP [41]. As for segmenting ground areas from hazardous obstacles, Wang adopted a mean-shift algorithm to regard the local point cloud to be a traversable area if the angle between the fitting plane and horizontal plane in the camera coordinate system is less than a threshold [42]. The approach achieves good robustness under certain environmental conditions with the reliance on thresholds and assumptions. Cheng put forward a seeded region growing algorithm to detect ground areas by adequately considering the depth boundaries instead of attaching importance to setting thresholds [11]. The approach withstands the fluctuations between frames but confuses VIP with the unstable results as the seeded pixels are randomly elected. Rodríguez estimated ground planes based on RANdom SAmple Consensus (RANSAC) and used a polar grid representation to account for the potential obstacles [27]. The approach involved a field test to verify the usability but it yielded a detection error in more than ten percent of the frames. Badino proposed to represent the 3D situation with a set of rectangular sticks for autonomous systems by taking into account the fact that the traversable area in front of vehicles is limited by objects with almost vertical surfaces and estimated the road by fitting a B-spline surface instead of assuming a planar road [43,44]. Elleuch developed a segmentation approach based on possibility modeling theory by imposing a crucial starting point, namely considering reference area placed in the bottom of the image to be traversable [45,46]. As for estimating the surface normal vector to determine traversable areas, Koester detected accessible sections by calculating the gradients and estimating the surface normal vector directions of real-world scene patches. The approach delivers fast and effective detection even in crowded scenes, but it prevents practical applications for user studies due to its overreliance on the quality of 3D reconstruction process and adherence to constraints [47]. For example, it assumes that area directly in front of the user is accessible. Bellone defined a descriptor to measure the unevenness of a local surface based on the estimation of normal vectors [48]. The index gives an enhanced description of the traversable area to perform obstacle avoidance and terrain navigability analysis simultaneously. However, it computes both the inclination and roughness at a low framerate which remains unpromising for assisting at normal walking speed. Ni proposed to extract safe directions by compressing the depth image from a light-coding sensor in the walking assistive robotic system which also comprises ultrasonic sensors to detect the evenness of road surfaces [49]. Cui predicted traversability based on support vector machine (SVM), and planned the optimal path for field robot with consideration of the travelling smoothness [50].

Several related work have been devoted to water hazard detection by taking multi-cue approaches with color, texture, depth and polarization information. Rankin detected water areas by analyzing brightness and saturation values, and searched for the reflections of ground cover with range information, and also discussed the possibility to specifically use the zero disparity to encode water area [51]. Yao extracted the features of brightness and texture from the non-reflective regions with color cameras, and detected the reflected regions where negative height exists and disparity sudden changes with stereo cues [52]. Xie detected water hazards by calculating the polarization degree image and used a self-adaptive segmentation algorithm and a morphology filtering technique to label water regions [53]. The method has good performance in complex natural backgrounds, but it requires mechanically rotating the linear polarizing filter and taking images at different angles, which results in a tradeoff between convenience and effectivity. Yao combined traditional machine learning and mean-shift segmentation, which accurately detects not only common water hazards, but also identifies special water hazards that may have lots of ripples or low brightness [54]. Rankin firstly found the horizon line and constrained the search for water bodies to the region below the horizon. Thereby, this approach decreases the computational cost of water detection as well as reduces the probability of false water detection [55]. Shao used a line-structured light sensor to achieve all-day water puddle detection by measuring the deformation of active light strip [56]. Kim presented a methodology using a stereo camera and treated the detection of wet areas and puddles differently [57]. For the detection of wet areas, the polarization difference, graininess as well as gradient magnitude are combined using hypothesis verification. For the detection of puddles, the depth map obtained by the stereo camera is used to check whether depth changes abruptly around the puddles. Nguyen set up a ZED stereo camera on top of a car with polarizing films on the camera lenses and tracked water hazards based on the polarization and color variation of reflected light with consideration of the effect of polarized light from the sky [58].

Almost all reported works were conducted under ideal scenarios or cause intolerable side effects in navigational assistance for VIP. To the best of our knowledge, no previous work has addressed the detection of water hazards to help VIP to avoid stepping in water areas and no previous work has fully combined the detection of traversable area and the detection of water hazard. Our approach builds on the prior work and makes the breakthrough while the superiority is apparent in the following aspects:

- The approach is able to detect traversable area and water hazard simultaneously, which prevents colliding into obstacles and stepping in water areas during navigation.
- The approach takes into account of the attitude angles of the sensor, which enables accurate detection with the continuous movement of the wearable prototype during navigation.
- The approach combines the detection of water hazard with the detection of traversable area, which decreases the computing cost and reduces water area detection error in scenarios such as sky regions and edges of buildings.

## 3. Approach

**Figure 1:**
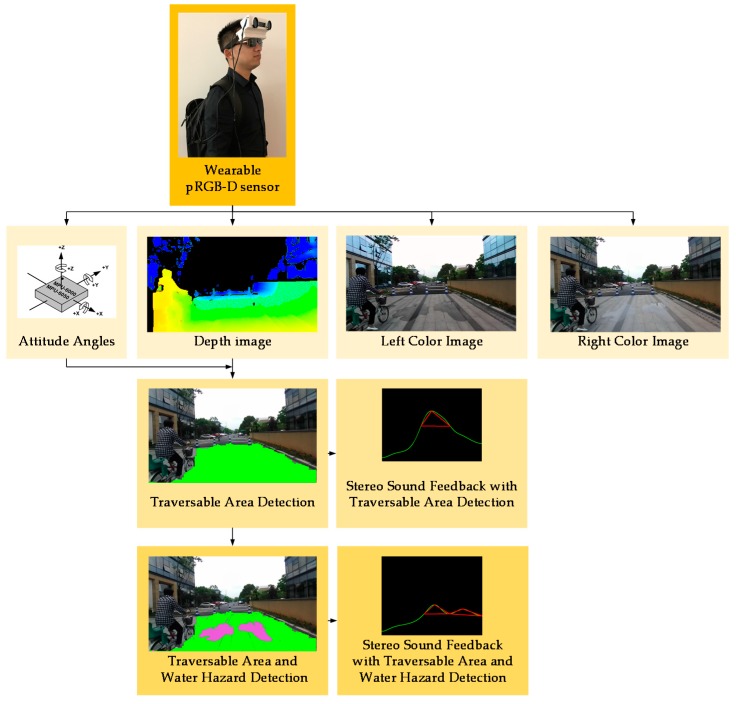
The flow chart of the approach.

In this section, the approach to detect traversable area and water hazard is elaborated in detail. As shown in Figure 1, the approach is described in terms of the pRGB-D sensor, the traversable area detection, the water hazard detection and the stereo sound feedback accordingly.

### 3.1. pRGB-D Sensor

As shown in Figure 1, the pRGB-D sensor is comprised of a ZED stereo camera which is equipped with horizontal and vertical polarization filters on the left and right camera, respectively, as well as a MPU6050 attitude sensor [59]. The stereo camera delivers 720 p left-right color video pairs and a depth image at 60 frames per second with the resolution same as the color video. The wide-angle all-glasses dual lenses share a diagonal field of view of 110 degrees. The sensor ranges from 0.5 m to 20 m which is quite suitable to detect traversable area and water hazard.

For the traversability awareness, it not only refers to long-range ground plane detection but also involves the early warning when a VIP approaches obstacles at close-range. For obstacle detection, the minimum range of 0.5 m, equivalent to the length of an arm, is enough for the avoidance of most obstacles given the normal walking speed of a VIP during navigation.

**Figure 2:**
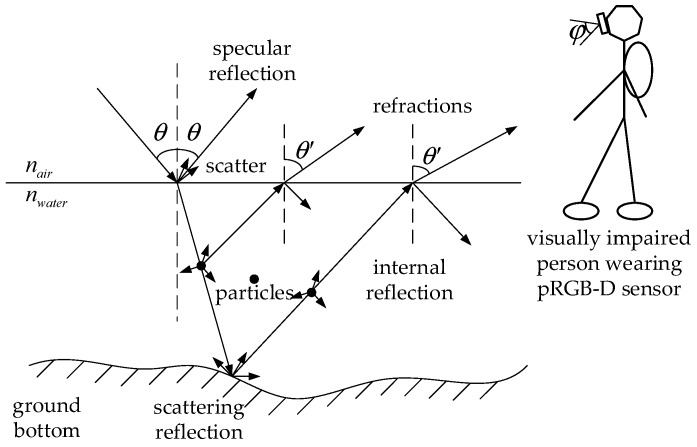
The light reflection and refraction for water hazards with particles and ground bottom.

For water hazard detection, the primary cue is the polarization effect as specular reflection on water is known to polarize light [58]. The specular reflection from the water surface $R_{reflect}$ is the sum of two polarization components $R_{reflect,⊥}$ and $R_{reflect,//}$, perpendicular and parallel respectively to the plane formed by the incident and reflected rays as given in Equations (1) and (2). As shown in Figure 2, $n_{air}$ and $n_{water}$ are the refractive indices of air and water respectively and θ is the reflection angle at the water surface.

(1) $$R_{reflect,⊥}(n_{air},n_{water},\theta )={\left[\frac{n_{air}cos\theta -n_{water}\sqrt{1-{\left(n_{air}/n_{water}\right)}^{2}sin^{2}\theta }}{n_{air}cos\theta +n_{water}\sqrt{1-{\left(n_{air}/n_{water}\right)}^{2}sin^{2}\theta }}\right]}^{2}$$

(2) $$R_{reflect,//}(n_{air},n_{water},\theta )={\left[\frac{-n_{water}cos\theta +n_{air}\sqrt{1-{\left(n_{air}/n_{water}\right)}^{2}sin^{2}\theta }}{n_{water}cos\theta +n_{air}\sqrt{1-{\left(n_{air}/n_{water}\right)}^{2}sin^{2}\theta }}\right]}^{2}$$

Suppose the polarized light from the air comprises energy components $E_{⊥}^{S}(\theta )$ and $E_{//}^{S}(\theta )$ for perpendicular and parallel components respectively as functions of reflection angle. The total energy entering the water can be calculated with Equation (3):

(3) $$F^{S}=E_{⊥}^{S}(\theta )[1-R_{reflect,⊥}(n_{air},n_{water},\theta )]+E_{//}^{S}(\theta )[1-R_{reflect,//}(n_{air},n_{water},\theta )]$$

However, part of the energy $F^{S}$ is scattered by suspended particles and ground bottom while the rest is absorbed by both particles and the ground as shown in Equation (4) where $\mu_{particles}$ and $\mu_{bottom}$ are the scattering coefficients of particles and the ground bottom respectively, and $\mu_{absorption}$ is the absorption coefficient:

(4) $$\mu_{particles}+\mu_{bottom}+\mu_{absorption}=1$$

Light in water can be considered as highly unpolarized light with random scattering and internal reflection. With part of the scattered light coming out of the water through refraction, the total light energy component coming out of the water is the summation of reflection and refraction for each polarization component, which can be calculated with Equations (5) and (6) where θ′ is the refraction angle from water to air:

(5) $$E_{⊥}^{R}(\theta )=E_{⊥}^{S}(\theta )R_{reflect,⊥}(n_{air},n_{water},\theta )+0.5F^{S}[\mu_{particles}+\mu_{bottom}]R_{refract,⊥}(n_{air},n_{water},\theta \prime =\theta )$$

(6) $$E_{//}^{R}(\theta )=E_{//}^{S}(\theta )R_{reflect,//}(n_{air},n_{water},\theta )+0.5F^{S}[\mu_{particles}+\mu_{bottom}]R_{refract,//}(n_{air},n_{water},\theta \prime =\theta )$$

**Figure 3:**
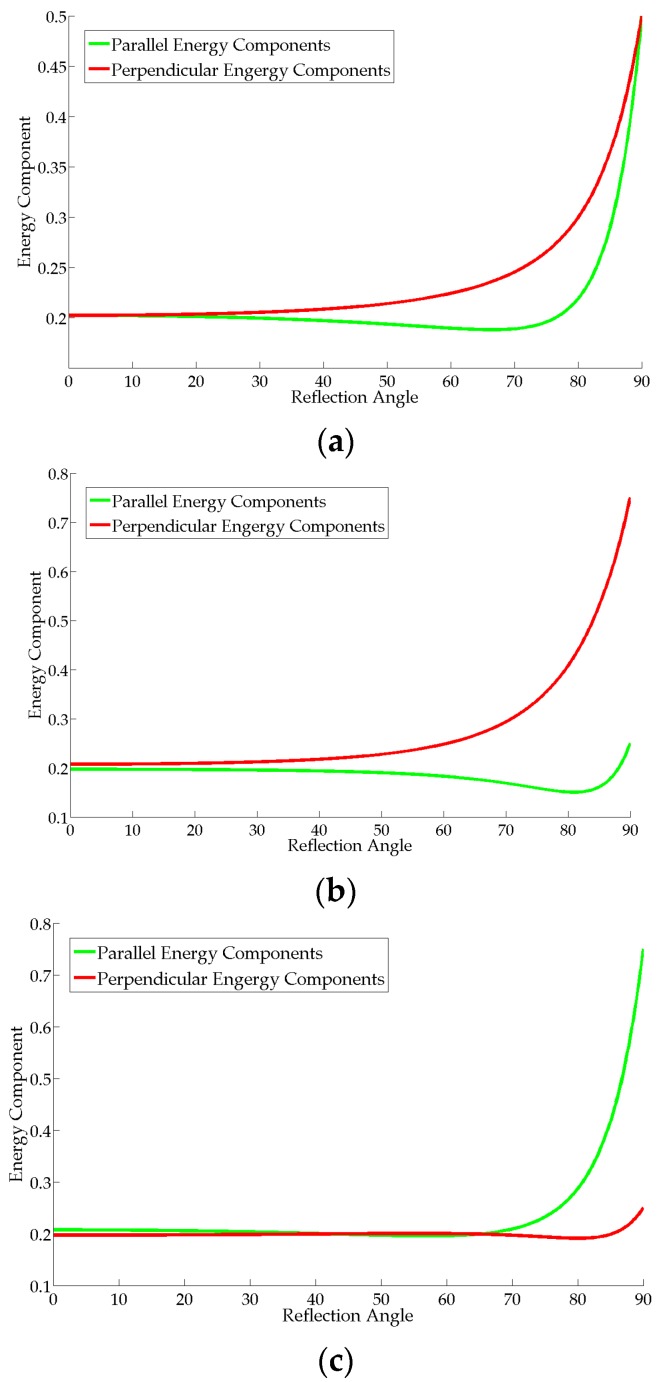
Reflection and refraction energy components from water as function of degree and direction of polarization. (**a**) Unpolarized light; (**b**) Light is three quarters polarized in perpendicular direction with the water plane; (**c**) Light is a quarter polarized in perpendicular direction with the water plane.

For illustrative purposes, in Figure 2, the water refractive index $n_{water}$ is set to 1.33 and the absorption coefficient $\mu_{absorption}$ is set to 60%. Figure 3 shows that the polarization difference between perpendicular and parallel components is large enough to provide a strong cue for water hazards at reflection angles above 70 degrees or at distances above the minimum detection range. Thereby, it is able to detect water hazards by setting the threshold of polarization difference with point correspondence from left color image to right color image.

If the wearing height of the pRGB-D sensor is H, the minimum water hazard detection range D is calculated with Equation (7), where φ is the vertical field view of the stereo camera, which equals 55.75 degrees:

(7) D=H×tan(θ−φ/2)

Thereby, given the average value of user height, the wearing height H is set to 1.6 m, and the reflection angle θ is set to 70 degrees. Thus, the minimum range D is around 1.45 m which is enough to provide immediate feedback to avoid stepping in water areas and quite matched with the working range of the depth sensor. However, with the continuous movement of the user head during navigation, the threshold to segment potential water hazards should be varying as the polarization direction of reflected light would not be strictly parallel or perpendicular with the polarizer in this circumstances. Given the rolling angle of the pRGB-D sensor equaling to η, which is obtained with an attitude sensor, the dynamic threshold is set to $\delta =\delta_{0}\cos^{2}\eta$, where $\delta_{0}$ is the threshold if there is no rolling. Additionally, notwithstanding different brightness of water area in left and right image due to the specular reflection, depth information of water areas on the road is available as the stereo camera in the pRGB-D sensor implemented a global stereo matching method to approximate smoothness constraint for compensating local brightness differences and radiometric transformations [35]. As a result, the pRGB-D sensor is quite suitable for navigational assistance thanks to its wearable computing technology and its compatibility to support traversable area and water hazard detection.

### 3.2. Traversable Area Detection

In order to detect traversable area, a simple and effective technique is presented. First, 3D coordinates of the point cloud are calculated. Given the depth Z of pixel (u,v) in the depth image, the calibrated focal length f, the radial distortion coefficients $c_{1}$, $c_{2}$, $c_{5}$, the tangential distortion coefficients $c_{3}$, $c_{4}$, as well as $\left(u_{0},v_{0}\right)$ the principal point, the point (X,Y,Z) in the camera coordinate system can be derived with Equations (8)−(13):

(8) $$x=\frac{u-u_{0}}{f}$$

(9) $$y=\frac{v-v_{0}}{f}$$

(10) $$r^{2}=x^{2}+y^{2}$$

(11) $$t=1+c_{1}r^{2}+c_{2}r^{4}+c_{5}r^{6}$$

(12) $$X=Z\times [xt+2c_{3}xy+c_{4}(r^{2}+2x^{2})]$$

(13) $$Y=Z\times [yt+2c_{4}xy+c_{3}(r^{2}+2y^{2})]$$

With the help of the attitude sensor, X, Y and Z coordinates in the camera coordinate system are adjusted to world coordinates. Assume a point in the camera coordinate system is (X,Y,Z) and the attitude angles acquired from the attitude sensor are (a,b,c). This means the point (X,Y,Z) rotates about the x-axis by α = a, then rotates about the y-axis by β = b and rotates about z-axis by γ = c in the end. Multiplying the point (X,Y,Z) by the rotation matrix given by Equation (14), the point $\left(X_{w},Y_{w},Z_{w}\right)$ in world coordinate system is obtained:

(14) $$\left[\begin{array}{c} X_{w}\\ Y_{w}\\ Z_{w} \end{array}\right]=\left[\begin{array}{ccc} \cos\gamma & -\sin\gamma & 0\\ \sin\gamma & \cos\gamma & 0\\ 0 & 0 & 1 \end{array}\right]\left[\begin{array}{ccc} \cos\beta & 0 & \sin\beta \\ 0 & 1 & 0\\ -\sin\beta & 0 & \cos\beta \end{array}\right]\left[\begin{array}{ccc} 1 & 0 & 0\\ 0 & \cos\alpha & -\sin\alpha \\ 0 & \sin\alpha & \cos\alpha \end{array}\right]\left[\begin{array}{c} X\\ Y\\ W \end{array}\right]$$

**Figure 4:**
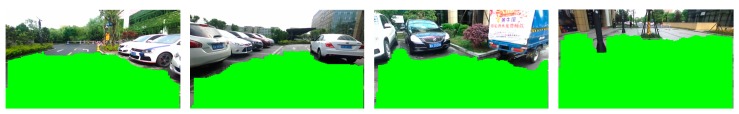
Traversable area detection in different scenarios.

After the point cloud adjustment, occupancy grids are computed with the stereo disparities transferred from the adjusted point cloud coordinates, and the method proposed in [60] is used to propagate the uncertainty of the stereo disparities onto the grid. In the polar occupancy grid, the image column is used to represent the angular coordinate and the adjusted disparity is used to represent the range. 3D points lying above the ground area are registered as obstacles in the occupancy grid and extremely high 3D points are dismissed such as ceilings which will not influence the travelling of VIP. Moreover, the ground pose is fitted by a B-Spline surface with consideration of two facts: real-world ground areas are not always planar surfaces [44] and the lenses share a distortion given a diagonal field of view of 110 degrees. Our system builds on the work of Stixel World [43], which represents the scene using a few upright objects on the ground plane. The computation includes dividing the input image into a set of vertical columns, and searching for regions that could be immediately reached without collision. In this sense, the task of traversable area detection is to find the first visible relevant obstacle in the positive direction of depth. In this work, instead of using a single threshold to segment obstacles and traversable areas independently, dynamic programming is used to seek the optimal path cutting the polar grid from left to right by imposing a spatial smoothness and a temporal smoothness to penalize both jumps in depth and deviations of the current frame from the previous frame. Figure 4 depicts frames of traversable area detection results by producing a green mask to overlay on the left color image acquired with the pRGB-D sensor.

### 3.3. Water Hazard Detection and Stereo Sound Feedback

**Figure 5:**
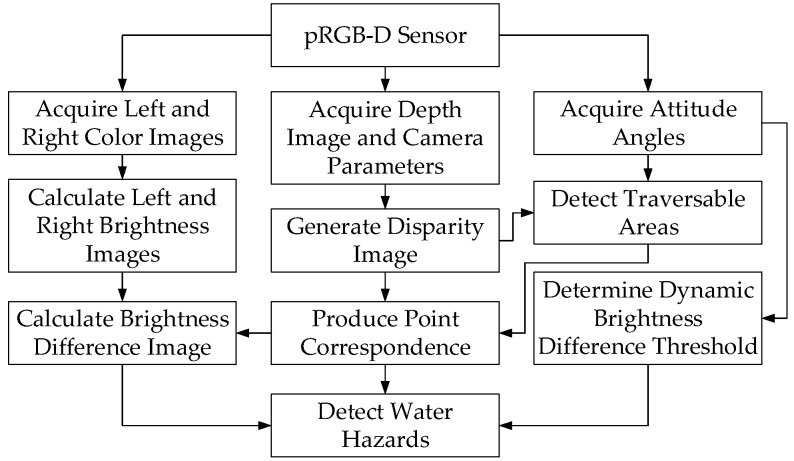
Flow chart of the water hazard detection.

As shown with the flowchart in Figure 5, in order to detect water hazards out of the traversable area, we first generate the disparity image from the depth image produced by the pRGB-D sensor.

Considering pixel G(u,v) in the depth image which ranges d, the calibrated focal length f, the baseline length B, the disparity value dis of pixel G can be calculated with Equation (15):

(15) dis=B×f/d

After that, we warp the right image to the left image to produce a point correspondence for water hazard detection. With the stereo pair, we are able to calculate a brightness difference image which signifies the polarization difference. Considering pixel G whose image coordinate is (u,v) in left image and corresponds to the pixel G′(u+dis,v) in right image. If the brightness at G(u,v) in left image is $V_{left}$ and the brightness in the warped right image is $V_{right}$ at G′(u+dis,v). The left image is captured by the left color camera with the horizontal polarizer while the right image is captured by the right color camera with the vertical polarizer. Thereby, the brightness difference of pixel G equals $\left|V_{left}-V_{right}\right|$. After extraction of the brightness difference image, it depends on the following conditions whether pixel G can be classified as water hazard:

- Pixel G is classified as traversable in the process as presented in Section 3.2, where traversable area is detected with depth and attitude information.
- $\left|V_{left}-V_{right}\right|>\delta$, where δ is the dynamic threshold of polarization difference set with the derivation in Section 3.1 and varies with the rolling angle of the pRGB-D sensor.

**Figure 6:**
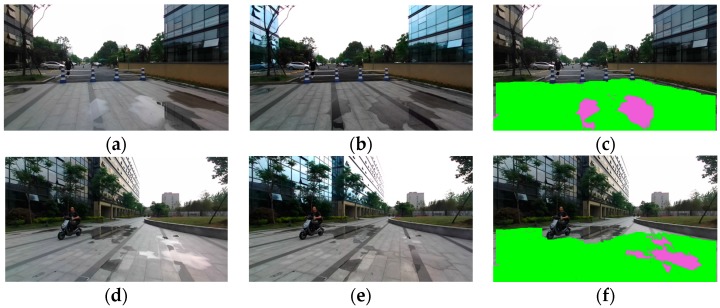
Water hazard detection with traversable area detection. (**a**,**d**) Left color images; (**b**,**e**) Right color images; (**c**,**f**) Detection results with the pRGB-D sensor.

Figure 6 depicts frames of water hazard detection results by producing magenta masks on the green traversable area detection results as presented in Section 3.2.

**Figure 7:**
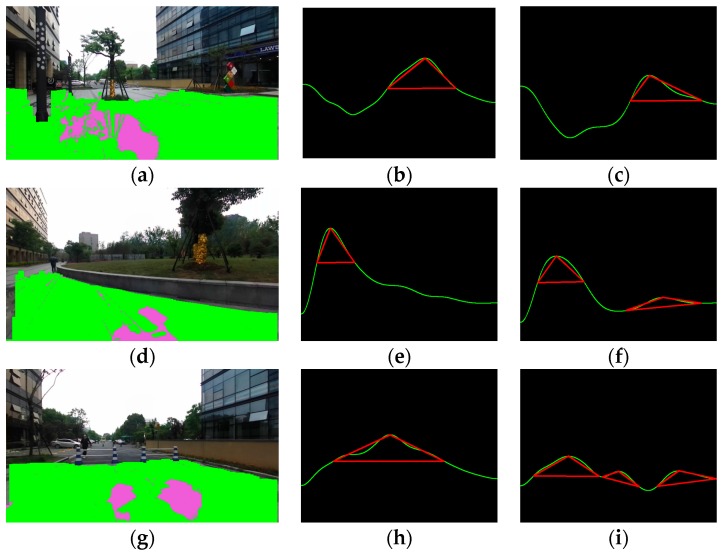
Farthest traversable lines with or without water hazard detection. (**a**,**d**,**g**) Traversable area and water hazard detection results; (**b**,**e**,**h**) Farthest traversable lines correspond to the traversable area detection without water hazard detection; (**c**,**f**,**i**) Farthest traversable lines obtained with both traversable area detection and water hazard detection.

After traversable area detection and water hazard detection, a farthest traversable line to represent the traversable distances in different directions is proposed to generate the stereo sound interface. In each direction, the farthest distance for navigating is determined by both the traversable area and water hazard detection results. In each direction, we first locate the farthest traversable pixel with the biggest depth value $Z_{t}$ and then search for the nearest pixel denoted as water area whose depth value is $Z_{w}$. The farthest traversable depth value $Z_{f}$ equals to $Z_{w}$ if a water area is detected and equals to $Z_{t}$ if no water areas are detected in this direction. Gaussian filtering is applied to smooth the farthest traversable line to reduce noises before generating feedback to VIP. After the filtering stage, the green farthest traversable lines with or without water hazard detection are obtained, as shown in Figure 7 where the red triangles represent the suggested navigation directions to take to avoid obstacles or water hazards.

This paper uses a variant of the non-semantic audio interface presented in our previous work [29] to transfer the traversable area and water hazard detection results to VIP by synthesizing stereo sound from the farthest traversable line. In this work, the mapping of traversable distances to the different sonification of instruments is elaborated.

**Figure 8:**
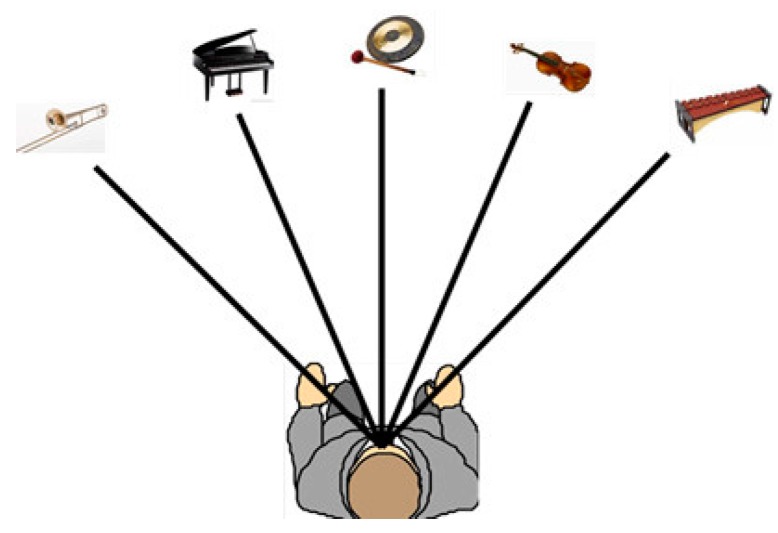
Stereo sound feedback of the assisting system. Sounds of five directions of traversable area with or without water hazard detection are presented by five musical instruments in the virtual 3D space, including trumpet, piano, gong, violin and xylophone.

As shown in Figure 8, the directions of traversable areas are differentiated not only by sound source locations in the virtual 3D space and the directions of stereo sound, but also by musical instruments, whose timbre differs from each other.

**Figure 9:**
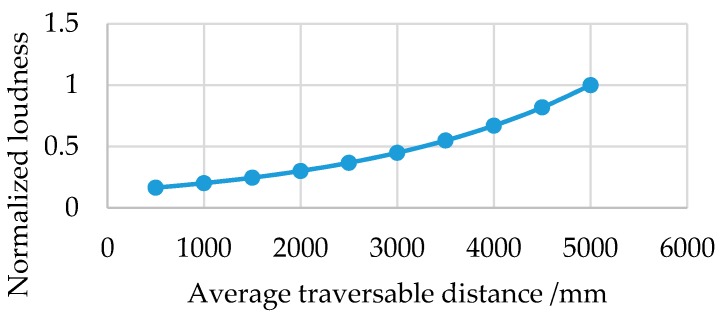
The relationship between the average traversable distance and instrument loudness.

**Figure 10:**
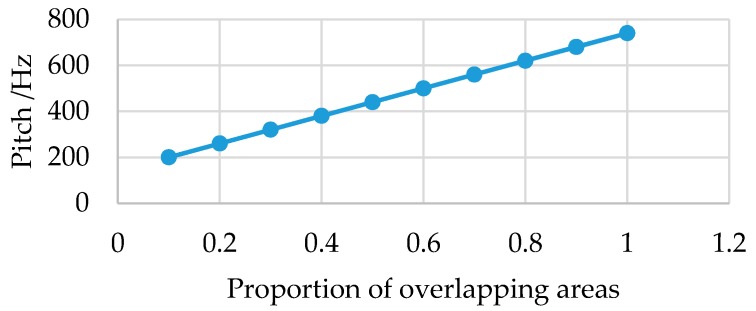
The relationship between the proportion of overlapping areas and instrument pitch.

The generation of the stereo sound follows rules below to guide and attract VIP to take the prioritized direction to detour around hazardous regions:

- Divide the farthest traversable line into five sections which correspond to the five different musical instruments. We only use five instruments to make sure that it is easy to understand and would not sound confusing after the familiarization period. Five instruments, including trumpet, piano, gong, violin and xylophone, produce sounds simultaneously.
- The horizontal field view of the pRGB-D sensor is 86.5°, so each musical instrument corresponds to the traversable line with a range of 17.3°.
- Each direction of traversable area and water hazard is represented by a musical instrument in the virtual 3D space.
- For each musical instrument, the bigger the sum of height in the corresponding section of the traversable line, the louder the sound from the instrument. As the relationship shown in Figure 9, the instrument loudness increases exponentially with the average traversable distance in the corresponding section of the traversable line so as to guide or attract the user to navigate the traversable direction.
- For each musical instrument and the corresponding section, the bigger the areas that overlap with the red triangle sections which denote suggested directions to take on navigation, the higher the pitch of the instrument. As the relationship shown in Figure 10, the instrument pitch increases linearly with the proportion of the overlapping areas with the red triangle sections.

## 4. Experiments

In this section, the proposed approach is verified with a series of experiments including tests on a score of indoor and outdoor scenarios, comparisons with other works in the literature and a field test in the real-world environment involving ten voluntary blindfolded users.

### 4.1. Indoor and Outdoor Detection Results

**Figure 11:**
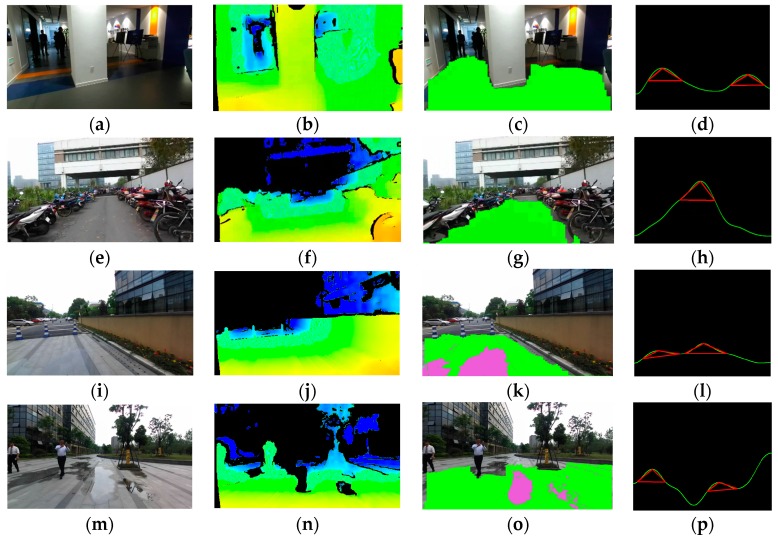
Results of traversable area and water hazard detection in indoor and outdoor environments. (**a**,**e**,**i**,**m**) Left color images; (**b**,**f**,**j**,**n**) Depth images; (**c**,**g**,**k**,**o**) Traversable area and water hazard detection results denoted respectively by the green and magenta masks on the left color images; (**d**,**h**,**l**,**p**) Farthest traversable lines as stereo sound feedback interface.

Figure 11 shows a number of traversable area detection and water area detection results in indoor and outdoor environments. The approach detects traversable areas and generates farthest traversable lines correctly, whose red triangles suggest the prioritized directions to avoid close obstacles and water hazards.

### 4.2. Comparison of Detection Results

**Figure 12:**
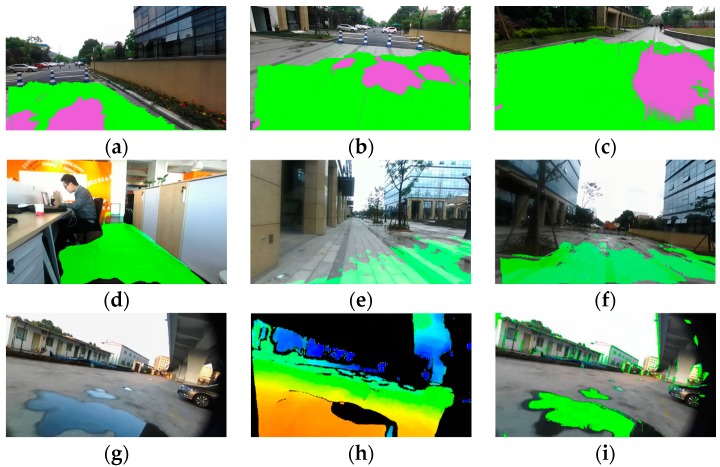
The detection results of different approaches. (**a**–**c**) Results of the proposed approach; (**d**–**f**) Results of traversable area detection based on RANSAC plus filtering techniques; (**g**–**i**) Results of water hazard detection based on sky light effect modelling.

To compare the performance of traversable area and water hazard detection with respect to other works, the results of several approaches are shown in Figure 8. Figure 12a−c are the results of the proposed approach which detects ground areas and water areas correctly. Comparatively, Figure 12d–f show the indoor and outdoor results of the approach proposed by Rodríguez which estimated the ground plane based on RANSAC plus filtering techniques [27]. Figure 12f is a correct result of detecting ground with some noises and holes, but the ground is partly detected in Figure 12e which is a type of sample error as the inclination angle of the plane is not considered.

We removed this kind of error in our work with adequate use of the attitude angles of the pRGB-D sensor. The approach in [58] set up a stereo camera to track water areas by modeling the sky light effect with reflection and azimuth angles. That approach is able to detect water hazards robustly, but mistakenly detects sky regions or edges of buildings as water areas as shown in Figure 12g−i. As the approach proposed in this paper combines the detection of water hazards and traversable areas, the aerial detection errors which would not hinder the navigation of VIP are reduced in number. Thus, the aerial errors would not be transferred to mislead VIP to avoid the fake water hazards. The total computation time of a single frame is 70.5 ms, while the image acquisition from the pRGB-D sensor takes 3 ms, and the time cost for the segmentation of traversable area and the detection of water hazard are 48 ms and 19.5 ms, respectively. Thereby, the computation cost is saved to maintain a reasonably qualified refresh rate of 14.2 frames per second on a 4.0 GHz Intel Core 7 processor with the resolution empirically set at 640 × 360, which is enough to feed results back in time to assist VIP at normal walking speed. 

In order to provide a quantitative evaluation of the approach, the performance of the algorithm is thoroughly analyzed by calculating traversable area detection rate (TADR), water area detection rate (WADR) and expansion error (EE). Given Equations (16)−(18), traversable area detection rate (TADR) is defined as the number of frames which ground has been detected correctly (GD) divided by the number of frames with ground (G). Water area detection rate (WADR) is defined as the number of frames which water area has been detected correctly (WAD) divided by the number of frames with water area (WA). Meanwhile, expansion error (EE) is defined as the number of frames which traversable area has been expanded to non-ground areas (ENG) including water areas divided by the number of frames with ground (G):

(16) $$TADR=\frac{GD}{D}$$

(17) $$WADR=\frac{WAD}{WA}$$

(18) $$EE=\frac{ENG}{G}$$

For quantitative analysis we used a sequence of 1285 frames from the pRGB-D dataset [61], which is built for tuning parameters and testing. The dataset is captured by the wearable sensor whose images of a single frame are respectively the depth image, left and right color images acquired with different polarizers whose polarization directions are perpendicular to each other after calibrations using a polarization analyzer. As shown in Table 1, the approach has a high traversable area detection rate of 94.4% compared with respect to other works [27,29]. Moreover, the expansion error has been decreased to 7.3% after the combination of traversable area detection and water area detection, illustrating the reliability of the approach.

### 4.3. Detection Limitations

**Figure 13:**
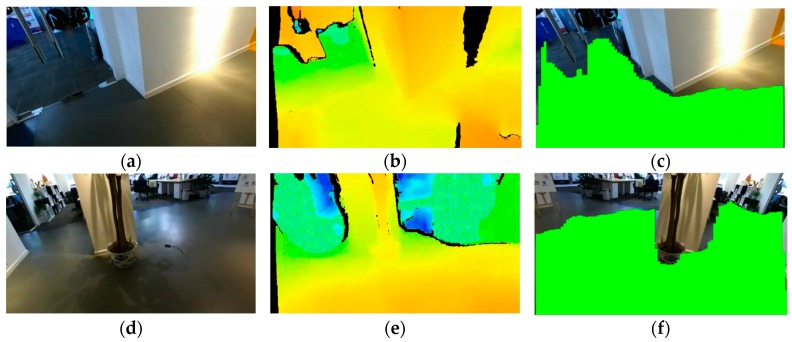
Detection limitations. (**a**–**c**) Detection with defective depth information; (**d**–**f**) Small obstacles would be detected as traversable area.

The proposed pipeline effectively combines polarization-color-depth-attitude information and improves the detection performance in indoor and outdoor environments, which is able to provide real-time assistances for VIP to avoid colliding into obstacles or stepping into water areas. However, it still has certain potential limitations. Since the proposed traversable area detection is based on the pRGB-D sensor whose depth information is acquired with stereo matching between left and right RGB images, the approach will have a slight problem when the depth information is defective as shown in Figure 13. As the textures of glass door and the overexposed region are poor, the depth information are invalid or filled with wrong values offered by the stereo type of RGB-D sensor in our prototype as shown in Figure 13a−c. Thereupon, the approach wrongly detects the region of glass door as a traversable area, and merely part of the ground area close to the overexposed wall is detected. Besides, as shown in Figure 13d–f, the approach is insensitive to small obstacles on the ground due to the depth filtering and dynamic programming strategy. To solve this problem, in the future time we aim to implement a small obstacle discovery algorithm [62] or provide both physical support and navigational indication for tiny threat awareness [49].

### 4.4. User Study

**Figure 14:**
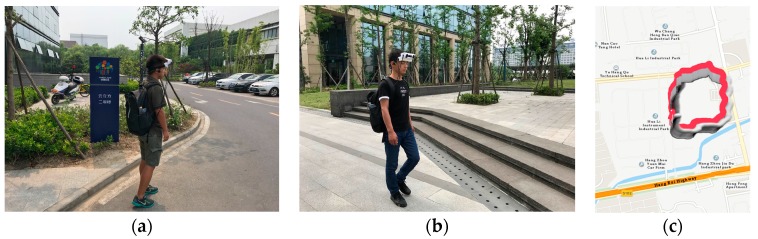
(**a**,**b**) The voluntary participants wearing the assisting prototype including a pRGB-D sensor, a laptop in the backpack and a set of bone-conduction headphone; (**c**) The route around a building and an empty field in the real-world environment while the red labels represent the locations during navigation.

To evaluate the ability of our system to help visually impaired users follow a prioritized route without losing their orientation or colliding into obstacles or stepping into water puddles, we conducted a user study in a real-world outdoor space. Before the participants were blindfolded, the stereo sound feedback of the system when wearing a bone-conduction headphone was explained as shown in Figure 14a,b. As we know, VIP or blindfolded users rely on cues from the environment a lot. For example, they use the sounds from their ears to understand the orientations of streets. Thereby, the assisting prototype is not only wearable but also ears-free, because the bone-conducting interface will not block the ears of participants from hearing environmental sounds. This familiarization period lasted for about seven minutes, during which they were able to adapt to hearing the stereo sound to keep away from close hazards including obstacles and water areas.

After the learning and familiarization stage, ten voluntary participants were blindfolded and were required to travel around a building and an empty field without colliding into obstacles or stepping into water areas. As a comparison task, the ten volunteers were divided into two groups randomly and while one group travelled with only traversable area detection, the other group travelled with both traversable area and water hazard detection. To provide the participants with a sense of orientation, the user would get an extra hint to turn at the bends or the road intersections. For a single test, three measures were recorded including the time needed by a participant to walk the route as shown in Figure 14c, the number of collisions into obstacles and the number of times they stepped into water areas. The route was acquired through recording the real-time locations during navigation with the red labels on the map provided by AMAP [63]. The timer starts when a participant was sent to the start region and stops when the participant completed navigating the route. As shown in Table 2, average number of times stepping into water areas with water hazard detection is significantly less than that without water hazard detection. It is the detection of water hazard in advance that endows participants to take the prioritized direction to avoid water hazards compared with the condition with only traversable area detection. Moreover, the average number of collisions into obstacles is few and most of the collisions occurred when the participants swerved and hit a curb which was outside the horizontal field view of the pRGB-D sensor. Comparatively, times of stepping in water areas are very few but not none, which is due to the minimum range of water hazard detection. If the user did not turn when hearing the stereo sound and kept moving forward, the water area would be out of the vertical field view of the pRGB-D sensor or the reflection angles would not be large enough to provide a strong cue for water hazards. Nonetheless, explicit feedback about directions to walk to avoid obstacles and water hazards were found helpful for assisting VIP. The results suggest that participants were aware of obstacles and water areas with our system and could make use of the stereo sound to keep away from or detour around the hazards. In other word, the safety and robustness of navigating VIP has been dramatically enhanced with the traversable area and water hazard detection based on the proposed pRGB-D framework.

After the test, the ten participants were asked two simple questions including whether the prototype was easy to wear and whether the stereo sound feedback could be used to find traversable directions. As shown in the questionnaire results (Table 3), all users answered that the system is useful and could help them to find traversable areas and avoid obstacles or water hazards.

Most participants were able to understand the changing process of loudness and pitch, and follow the stereo sound to find the traversable directions and detour around the hazards, while a small group of participants only used the direction of the stereo sound to turn left or turn right to avoid collisions. This demonstrated the usefulness and effectivity of the approach in terms of traversable area detection and stereo sound feedback. In addition, some users provided some advice on adding functions, such as the detection of curbs and negative obstacles. Moreover, the users were optimistic about the pRGB-D system and would like to have a more profound experience.

## 5. Conclusions

RGB-D sensors are a ubiquitous choice to assist navigation for the visually impaired. However, most solutions are confined to using intensity information whereas the polarization cues are weak. The proposed pRGB-D framework enhances the safety and robustness of navigation by combining traversable area detection and water hazard detection with polarization-color-depth-attitude information. Indoor and outdoor empirical evidences, quantitative analysis of the detection as well as a user study with ten blindfolded participants demonstrated its usefulness and reliability.

In the future, we will improve our navigation assistance approach to reach a higher level of perception and offer more independence to visually impaired individuals. Specifically, we look forward to including polarization for ranging and further investigating discovery schemes for the detection of small obstacles such as curbs and transparent objects. Additionally, it is necessary to run a larger study with visually impaired participants to test this approach, and different audio output settings could be compared. The use of portable processors and sensors with larger field of view would also benefit the system in terms of wearable mobility and detectable performance.

## Figures and Tables

**Table 1:** Quantitative analysis.

Approaches	TADR%	WADR%	EE%
Proposed approach without water area detection	94.4%	N/A	60.6%
Proposed approach with water area detection	94.4%	89.2%	7.3%
RANSAC plus filtering techniques [27]	79.6%	N/A	41.5%
RANSAC plus expanding techniques [29]	93.8%	N/A	65.2%
3D tracking of water areas [58]	N/A	86.5%	N/A

**Table 2:** Average time, number of collisions and number of times stepping in water areas in two conditions: the stereo sound interface transferred to the participants is generated according to the traversable area detection result with/without water hazard detection.

Transferred Detection Result	Average Time to Complete	Average Number of Collisions into Obstacles	Average Number of Times Stepping in Water Areas
traversable area detection	24 min 32 s	3.0	30.6
traversable area and water hazard detection	26 min 11 s	2.6	2.8

**Table 3:** Questionaire. After the field test, ten participants were asked two simple questions.

User	Easy to Wear?	Could You Use the Stereo Sound Feedback to Find Traversable Directions?
User 1	Yes	Yes
User 2	Yes	Yes
User 3	No	Yes
User 4	Yes	Yes
User 5	Yes	Yes
User 6	Yes	Yes
User 7	No	Yes
User 8	No	Yes
User 9	Yes	Yes
User 10	Yes	Yes

## References

[1] World Health Organization Visual Impairment and Blindness. http://www.who.int/mediacentre/factsheets/fs282/en/.

[2] Zollner M., Huber S., Jetter H., Reiterer H.C., Reiterer H., Graham P.C.N., Nunes J.J.N., Winckler P.M.M. (2007). NAVI—A Proof-of-Concept of a Mobile Navigational Aid for Visually Impaired Based on the Microsoft Kinect. Human—Computer Interaction—INTERACT 2011.

[3] Takizawa H., Yamaguchi S., Aoyagi M., Ezaki N., Mizuno S. (2012). Kinect cane: An assistive system for the visually impaired based on three-dimensional object recognition. Pers. Ubiquitous Comput..

[4] Filipe V., Fernandes F., Fernandes H., Sounsa A., Paredes H., Barroso J. Assisted Guidance for the Blind Using the Kinect Device. Proceedings of the 7th International Conference on Software Development and Technologies for Enhancing Accessibility and Fighting Info-exclusion.

[5] Park C.H., Howard A.M. Real-time haptic rendering and haptic telepresence robotic system for the visually impaired. Proceedings of the World Haptics Conference (WHC).

[6] Hicks S.L., Wilson I., Muhammed L., Worsfold J., Downes S.M., Kennard C. (2013). A depth-based head-mounted visual display to aid navigation in partially sighted individuals. PLoS ONE.

[7] Wang Z., Liu H., Wang X., Qian Y. Segment and label indoor scene based on RGB-D for the visually impaired. Proceedings of the International Conference on Multimedia Modeling.

[8] Aladren A., Lopez-Nicolas G., Puig L., Guerrero J.J. (2014). Navigational assistance for the visually impaired using rgb-d sensor with range expansion. IEEE Syst. J..

[9] Hsieh C.T., Lai W.M., Yeh C.H., Huang H.C. (2013). An Obstacle Detection System Using Depth Information and Region Growing for Blind. Res. Notes Inf. Sci..

[10] Guerrero J.J., Pérez-Yus A., Gutiérrez-Gómez D., Rituerto A., López-Nicolás G. Human navigation assistance with a RGB-D sensor. Proceedings of the ACTAS V Congreso Internacional de Turismo para Todos: VI Congreso Internacional de Diseño, Redes de Investigación y Tecnología para todos DRT4ALL.

[11] Cheng R., Wang K., Yang K., Zhao X. A ground and obstacle detection algorithm for the visually impaired. Proceedings of the IET International Conference on Biomedical Image and Signal Processing (ICBISP 2015).

[12] Yang K., Wang K., Cheng R., Zhu X. A new approach of point cloud processing and scene segmentation for guiding the visually impaired. Proceedings of the IET International Conference on Biomedical Image and Signal Processing (ICBISP 2015).

[13] Saputra M.R.U., Santosa P.I. Obstacle Avoidance for Visually Impaired Using Auto-Adaptive Thresholding on Kinect’s Depth Image. Proceedings of the 2014 IEEE 11th Intl Conf on Ubiquitous Intelligence and Computing and 2014 IEEE 11th Intl Conf on Autonomic and Trusted Computing and 2014 IEEE 14th International Conference on Scalable Computing and Communications and Its Associated Workshops.

[14] Blessenohl S., Morrison C., Criminisi A., Shotton J. Improving Indoor Mobility of the Visually Impaired with Depth-Based Spatial Sound. Proceedings of the IEEE International Conference on Computer Vision Workshops.

[15] Perez-Yus A., Lopez-Nicolas G., Guerrero J.J. Detection and modelling of staircases using a wearable depth sensor. Proceedings of the European Conference on Computer Vision.

[16] Munoz R., Rong X., Tian Y. Depth-aware indoor staircase detection and recognition for the visually impaired. Proceedings of the 2016 IEEE International Conference on Multimedia & Expo Workshops (ICMEW).

[17] Perez-Yuz A., Gutierrez-Gomez D., Lopez-Nicolas G., Guerrero J.J. (2017). Stairs detection with odometry-aided traversal from a wearable RGB-D camera. Comput. Vis. Image Underst..

[18] Wong F., Nagarajan R., Yaacob S. Application of stereovision in a navigation aid for blind people. Proceedings of the 2003 IEEE Joint Conference of the Four Information Communications and Signal Processing, and Fourth Pacific Rim Conference on Multimedia.

[19] Johnson L.A., Higgins C.M. A navigation aid for the blind using tactile-visual sensory substitution. Proceedings of the 28th Annual International Conference of the IEEE on Engineering in Medicine and Biology Society (EMBS’06).

[20] Rodriguez A., Bergasa L.M., Alcantarilla P.F., Yebes J., Cela A. Obstacle avoidance system for assisting visually impaired people. Proceedings of the IEEE Intelligent Vehicles Symposium Workshops.

[21] Martinez J.M.S., Ruiz F.E. Stereo-based aerial obstacle detection for the visually impaired. Proceedings of the Workshop on Computer Vision Applications for the Visually Impaired.

[22] Pradeep V., Medioni G., Weiland J. Robot vision for the visually impaired. Proceedings of the 2010 IEEE Computer Society Conference on Computer Vision and Pattern Recognition Workshops (CVPRW).

[23] Brilhault A., Kammoun S., Gutierrez O., Truillet P., Jouffrais C. Fusion of artificial vision and GPS to improve blind pedestrian positioning. Proceedings of the 2011 4th IFIP International Conference on New Technologies, Mobility and Security (NTMS).

[24] Lee Y.H., Medioni G. RGB-D camera based navigation for the visually impaired. Proceedings of the RSS RGBD Advanced Reasoning with Depth Camera Wproorkshop.

[25] Alcantarilla P.F., Yebes J.J., Almazan J., Bergasa L.M. On Combining visual SLAM and dense scene flow to increase the robustness of localization and mapping in dynamic environments. Proceedings of the 2012 IEEE International Conference on Robotics and Automation (ICRA).

[26] Lin K.W., Lau T.K., Cheuk C.M., Liu Y. A wearable stereo vision system for visually impaired. Proceedings of the 2012 International Conference on Mechatronics and Automation (ICMA).

[27] Rodriguez A., Yebes J.J., Alcantarilla P.F., Bergasa L.M., Almazan J., Cele A. (2011). Assisting the visually impaired: Obstacle detection and warning system by acoustic feedback. Sensors.

[28] Miksik O., Vineet V., Lidegaard M., Prasaath R., Nebner M., Golodetz S., Hicks S.L., Pérez P., Izadi S., Torr P.H.S. The semantic paintbrush: Interactive 3d mapping and recognition in large outdoor spaces. Proceedings of the 33rd Annual ACM Conference on Human Factors in Computing Systems.

[29] Yang K., Wang K., Hu W., Bai J. (2016). Expanding the Detection of Traversable Area with RealSense for the Visually Impaired. Sensors.

[30] Huang X., Bai J., Wang K., Liu Q., Luo Y., Yang K., Zhang X. (2017). Target enhanced 3D reconstruction based on polarization-coded structured light. Opt. Express.

[31] Ryan Fanello S., Rhemann C., Tankovich V., Kowdle A., Orts Escolano S., Kim D., Izadi S. Hyperdepth: Learning depth from structured light without matching. Proceedings of the IEEE Conference on Computer Vision and Patten Recognition.

[32] Einecke N., Eggert J. A two-stage correlation method for stereoscopic depth estimation. Proceedings of the 2010 International Conference on Digital Image Computing: Techniques and Applications (DICTA).

[33] Einecke N., Eggert J. Block-matching stereo with relaxed fronto-parallel assumption. Proceedings of the Intelligent Vehicles Symposium.

[34] Einecke N., Eggert J. A multi-block matching approach for stereo. Proceedings of the 2015 IEEE Intelligent Vehicles Symposium (IV).

[35] Hirschmuller H. (2008). Stereo processing by semiglobal matching and mutual information. IEEE Trans. Pattern Anal. Mach. Intell..

[36] Xie Y., Zeng S., Chen L. A Novel Disparity Refinement Method Based on Semi-Global Matching Algorithm. Proceedings of the 2014 IEEE International Conference on Data Mining Workshop (ICDMW).

[37] Hernandez-Juarez D., Chacon A., Espinosa A., Vazquez D., Moure J.C., Lopez A.M. (2016). Embedded real-time stereo-estimation via Semi-Global Matching on the GPU. Procedia Comput. Sci..

[38] Keselman L., Woodfill J.I., Grunnet-Jepsen A., Bhowmik A. (2017). Intel RealSense Stereoscopic Depth Cameras. arXiv.

[39] Konolige K. Projected texture stereo. Proceedings of the 2010 IEEE International Conference on Robotics and Automation(ICRA).

[40] Stereolabs. Http://www.stereolabs.com.

[41] Dakopoulos D., Bourbakis N.G. (2015). Wearable obstacle avoidance electronic travel aids for blind: A survey. IEEE Trans. Syst. Man Cybern. Part C Appl. Rev..

[42] Wang T., Bu L., Huang Z. A new method for obstacle detection based on Kinect depth image. Proceedings of the 2015 Chinese Automation Congress (CAC).

[43] Badino H., Franke U., Pfeiffer D. The stixel world-a compact medium level representation of the 3D-world. Proceedings of the 31st DAGM Symposium.

[44] Wedel A., Franke U., Badino H., Cremers D. B-spline modelling of road surfaces for freespace estimation. Proceedings of the 2008 IEEE Intelligent Vehicles Symposium.

[45] Elleuch J.F., Bellaaj M., Sellami D., Kallel I.K. Travesable area segmentation approach at indoor environment for visually impaired people. Proceedings of the 13th International Conference on Advances in Mobile Computing and Multimedia.

[46] Frikha J., Sellami D., Kallel I.K. (2016). Indoor/outdoor navigation system based on possibilistic traversable area segmentation for visually impaired people. ELCVIA Electron. Lett. Comput. Vis. Imag. Anal..

[47] Koester D., Schauerte B., Stiefelhagen R. Accessible section detection for visual guidance. Proceedings of the IEEE International Conference on Multimedia and Expo Workshops.

[48] Bellone M., Messina A., Reina G. A new approach for terrain analysis in mobile robot applications. Proceedings of the IEEE International Conference on Mechatronics.

[49] Ni D., Song A., Tian L., Xu X., Chen D. (2015). A walking assistant robotic system for the visually impaired based on computer vision and tactile perception. Int. J. Soc. Robot..

[50] Cui J., Guo Y., Zhang H., Qian K., Bao J., Song A. (2013). Support Vector Machine Based Robotic Traversability Prediction with Vision Features. Int. J. Comput. Intell. Syst..

[51] Rankin A.L., Matthies L.H., Huertas A., Wei T.K. (2006). Daytime water detection by fusing multiple cues for autonomous off-road navigation. Transformational Science and Technology for the Current and Future Force.

[52] Yao T., Xiang Z., Liu J., Xu D. Multi-feature fusion based outdoor water hazards detection. Proceedings of the 2007 IEEE International Conference on Mechatronics and Automation (ICMA).

[53] Xie B., Pan H., Xiang Z., Liu J. Polarization-based water hazards detection for autonomous off-road navigation. Proceedings of the International Conference on Mechatronics and Automation (ICMA).

[54] Yao T.Z., Xiang Z.Y., Liu J.L. (2009). Robust water hazard detection for autonomous off-road navigation. J. Zhejiang Univ..

[55] Rankin A.L., Matthies L.H., Bellutta P. Daytime water detection based on sky reflections. Proceedings of the 2011 IEEE International Conference on Robotics and Automation (ICRA).

[56] Shao H., Zhang Z., Li K. Research on water hazard detection based on line structured light sensor for long-distance all day. Proceedings of the 2015 IEEE International Conference on Mechatronics and Automation (ICMA).

[57] Kim J., Baek J., Choi H., Kim E. (2016). Wet area and puddle detection for Advanced Driver Assistance Systems (ADAS) using a stereo camera. Int. J. Control Autom. Syst..

[58] Nguyen C.V., Milford M., Mahony R. (2017). 3D tracking of water hazards with polarized stereo cameras. arXiv.

[59] InvenSense MPU-6050. https://playground.arduino.cc/Main/MPU-6050.

[60] Badino H., Franke U., Mester R. Free space computation using stochastic occupancy grids and dynamic programming. Proceedings of the 2007 ICCV Workshop on Dynamical Vision.

[61] Kaiwei Wang Team. wangkaiwei.org.

[62] Kumar S., Karthik M.S., Krishna K.M. Markov Random Field based small obstacle discovery over images. Proceedings of the 2014 IEEE International Conference on Robotics and Automation (ICRA).

[63] AMAP. http://ditu.amap.com/.

